# Dysconnectivity of the medio-dorsal thalamic nucleus in drug-naïve first episode schizophrenia: diagnosis-specific or trans-diagnostic effect?

**DOI:** 10.1038/s41398-018-0350-0

**Published:** 2019-01-16

**Authors:** Qiyong Gong, Vaisakh Puthusseryppady, Jing Dai, Manxi He, Xin Xu, Yan Shi, Baiwan Zhou, Yuan Ai, Cheng Yang, Feifei Zhang, Su Lui, Andrea Mechelli

**Affiliations:** 1 Huaxi MR Research Center (HMRRC), Departments of Radiology, West China Hospital of Sichuan University, Chengdu, China; 2 Department of Psychoradiology, Chengdu Mental Health Center, Chengdu, China; 3 Department of Psychiatry, West China Hospital of Sichuan University, Chengdu, China; 4 Department of Psychosis Studies, Institute of Psychiatry, Psychology & Neuroscience, King’s College London, London, UK; 5 Norwich Medical School, University of East Anglia, Norwich, UK

## Abstract

Converging lines of evidence implicate the thalamocortical network in schizophrenia. In particular, the onset of the illness is associated with aberrant functional integration between the medio-dorsal thalamic nucleus (MDN) and widespread prefrontal, temporal and parietal cortical regions. Because the thalamus is also implicated in other psychiatric illnesses including post-traumatic stress disorder (PTSD) and major depressive disorder (MDD), the diagnostic specificity of these alterations is unclear. Here, we determined whether aberrant functional integration between the MDN and the cortex is a specific feature of schizophrenia or a trans-diagnostic feature of psychiatric illness. Effective connectivity (EC) between the MDN and rest of the cortex was measured by applying psychophysiological interaction analysis to resting-state functional magnetic resonance imaging data of 50 patients with first episode schizophrenia (FES), 50 patients with MDD, 50 patients with PTSD and 122 healthy controls. All participants were medication-naïve. The only significant schizophrenia-specific effect was increased EC between the right MDN and the right pallidum (*p* < 0.05 corrected). In contrast, there were a number of significant trans-diagnostic alterations, with both right and left MDN displaying trans-diagnostic increased EC with several prefrontal and parietal regions bilaterally (*p* < 0.05 corrected). EC alterations between the MDN and the cortex are not specific to schizophrenia but are a trans-diagnostic feature of psychiatric disorders, consistent with emerging conceptualizations of mental illness based on a single general psychopathology factor. Therefore, dysconnectivity of the MDN could potentially be used to assess the presence of general psychopathology above and beyond traditional diagnostic boundaries.

## Introduction

Schizophrenia, a severe psychiatric illness involving delusions, hallucinations and disorganized thinking, is one of the greatest causes of disability worldwide^1^. Over the past two decades, magnetic resonance imaging (MRI) techniques such as resting-state functional MRI (rs-fMRI) have allowed the investigation of the neurobiological alterations underlying this disease in vivo. These studies have revealed widespread alterations within cortical-subcortical-cerebellar networks^2^, and in particular have identified the thalamus as a key structure implicated in the onset and progression of the illness^3–6^. Within this region, the medio-dorsal thalamic nucleus (MDN) has received particular attention due to its prominent connections to the prefrontal cortex, a region known to be implicated in the illness^7^. Task-based studies have reported decreased activation in the MDN of patients during a range of cognitive processes including episodic memory, working memory and attention^8^, whilst resting-state studies have found aberrant functional integration between the MDN and widespread cortical networks including prefrontal, temporal, and parietal regions^9^.

In addition to schizophrenia, the thalamus has also been implicated in other psychiatric illnesses including post-traumatic stress disorder (PTSD) and major depressive disorder (MDD). In PTSD, thalamic functional abnormalities are thought to underlie dysregulation of sensory filtering, circadian rhythms, level of alertness and consciousness^10^. Moreover, with the thalamus being a fundamental component of the mood regulating circuit^11^, functional abnormalities in this region are thought to contribute to emotional dysregulation in MDD^12^.

A critical limitation of the existing literature is that the diagnostic specificity of the findings is unclear, since previous studies have compared a single group of patients with a certain diagnosis against a group of healthy controls. Additionally, studies have typically analyzed chronic patients under pharmacological treatment, raising the possibility that the observed alterations reflect a consequence of medication rather than an intrinsic feature of psychopathology. In particular, antipsychotic treatment represents a potential major confound in light of previous studies suggesting that it can influence resting-state thalamocortical functional integration^13^.

The aim of the present investigation was therefore to examine the diagnostic specificity of alterations in thalamocortical functional integration in schizophrenia without the confounding effects of medication. In light of the number of previous studies in patients with schizophrenia reporting alterations in the MDN specifically^3,7–9^, the present investigation focused on this section of the thalamus. By comparing effective connectivity alterations in medication-naïve patients with schizophrenia, PTSD, and MDD, we were able to differentiate between effects that are specific to psychotic illness and effects that represent a generic, trans-diagnostic feature of psychiatric disease. We used rs-fMRI in a total of 272 un-medicated participants including 50 with first episode schizophrenia (FES), 50 with a diagnosis of PTSD, 50 with a diagnosis of MDD and 122 healthy controls (HC). We hypothesized the presence of thalamocortical alterations specific to schizophrenia, reflecting the unique clinical presentation of this illness. Furthermore, we hypothesized the presence of trans-diagnostic thalamocortical alterations, reflecting shared psychopathological features of the three diseases including general psychopathology^14^. The results would inform current neurobiological models of psychiatric disease and support the development of differential biomarkers to help classify patients between alternative diagnoses in clinical practice.

## Materials and methods

### Participants

The study was approved by the Medical Ethics Committee of West China Hospital of Sichuan University and all participants provided written informed consent. Subsets of the data used here have been used in previous studies^15–18^. While in a recent article^18^ we consider functional integration within and between three networks of interest (default mode, central exectutive, and salience), in the present investigation we focus on the EC between the MDN and the cortex. All patients were recruited as they presented to the West China Hospital of Sichuan University, and were came from the inner city of Chengdu with the exception of the PTSD group who tended to come from surrounding rural areas (see below) Exclusion criteria applicable to all participants included (i) history of drug or alcohol abuse, (ii) pregnancy and (iii) any physical illness such as hepatitis, cardiovascular disease, or neurological disorder, as assessed by interview and review of medical records. Further information on each group are provided below.

### First episode schizophrenia

Fifty patients with FES were included (see Table 1). The presence of psychotic illness was determined by the consensus of two experienced psychiatrists using the Structured Interview for the DSM-IV Axis I Disorder, Patient Edition (SCID). Psychopathology was measured on the day of scanning using the Positive and Negative Syndrome Scale (PANSS)^19^. The use of these clinical instruments indicated that all patients met criteria for schizophrenia—a diagnosis that was confirmed in subsequent clinical follow-ups. At the time of scanning, all patients were medication-naïve.

**Table 1:** Characteristics of subject cohorts

Measure	FES			PTSD			MDD			HC		
	(*n* = 50)			(*n* = 50)			(*n* = 50)			(*n* = 122)		
	*n*	Mean	SD	*n*	Mean	SD	*n*	Mean	SD	*n*	Mean	SD
Age	–	32.72	7.27	–	43.10	10.79	–	38.89	11.63	–	29.61	14.36
Gender (M:F)	16:34	–	–	16:34	–	–	16:34	–	–	58:64	–	–
Education years	–	13.28	4.12	–	6.90	3.44	–	12.83	3.93	–	12.33	3.08
PANSS positive		24.84	6.86									
PANSS negative	–	16.84	8.66	–	–	–	–	–	–	–	–	–
PANSS general		46.62	9.35									
PANSS total		88.30	19.61									
PCL	–	–	–	–	51.20	10.44	–	–	–	–	–	–
HAM-D	–	–	–	–	–	–	–	23.22	4.53	–	–	–

*PANSS* positive and negative syndrome scale, *PCL* PTSD Checklist, HAM-D Hamilton depression rating scale, *SD* standard deviation, *M* males, F females

### Major depressive disorder

Fifty participants with MDD were included (see Table 1). These participants were part of a larger cohort study of depression in the Chinese population of Han nationality. Diagnosis of MDD was made with the SCID; although psychotic symptoms can occur during depressive episodes, none of the participants reported suffering from them. On the day of scanning, severity of depression was quantified using the 17-item Hamilton depression rating scale (HAM-D)^20^ and all participants had a total HAM-D score ≥18. All patients were medication-naïve at the time of scanning.

### Post-traumatic stress disorder

Fifty participants with PTSD were included^17^ (see Table 1). These participants were survivors of the 2008 Sichuan earthquake who lived in rural areas, had all physically experienced the earthquake, personally witnessed death, serious injury or the collapse of buildings and not suffered any physical injury; because these patients were recruited in the aftermath of the earthquake, they came from rural areas (where the Earthquake was strongest) and were older relative to the other clinical groups. All participants were assessed using the PTSD Checklist (PCL)^21^ and met threshold criteria (PCL score ≥ 35) for diagnosis of PTSD on the day of scanning. At the time of scanning, all patients were medication-naïve.

### Healthy controls

One hundred and twenty two HC were included (Table 1). All were recruited by poster advertisement, and screened using the non-patient edition of the SCID to confirm the lifetime absence of psychiatric illnesses. In addition, all were interviewed to exclude individuals with a known history of psychiatric illness in first-degree relatives.

### Data acquisition

All neuroimaging data was acquired from a 3T MRI scanner (EXCITE; General Electric, Milwaukee, Wisconsin) with an 8-channel phased array head coil. Images were acquired using a gradient-echo echo-planar imaging sequence with a repetition time (TR) = 2000 ms, an echo time (TE) = 30 ms, flip-angle = 90°, slice thickness = 5 mm (no slice gap), 64 × 64 matrix and field of view = 24 cm^2^ resulting in a voxel size of 3.75 × 3.75 × 5 mm^3^. Scanning lasted for 410 s, with each brain volume comprising 30 axial slices and each functional run containing 205 image volumes. During scanning, participants were instructed to relax with their eyes closed without falling asleep. After the experiment, each participant confirmed as to not having fallen asleep during scanning.

### Data analysis

#### Preprocessing

Functional images were pre-processed using SPM12 software (http://www.fil.ion.ucl.ac.uk/spm) running under Matlab 7.1 (Math Works, USA). For each participant, the first five scans were discarded to remove the impact of magnetization stabilization, and the images were realigned to the first of the run and resliced with sinc interpolation. The resulting images were normalized to the Montreal Neurological Institute (MNI) 152 template and then linearly de-trended and band pass filtered (0.01–0.08 Hz) to remove low-frequency drift and high-frequency physiological noise. Finally the normalized images were smoothed with an 8-mm full width at half maximum isotropic Gaussian kernel, and the global signal, the white matter signal and the cerebrospinal fluid signal were regressed out. None of the subjects who enrolled on the study had to be excluded due to excessive head motion during scanning (defined as translational movement >1.5 mm and/or rotation >1.5°). In order to test for differences in head movement, we estimated the framewise displacement of each subject—a measure of head movement from one volume to the next calculated as the sum of the absolute values of the realignment estimates at every time point^22^; an ANOVA did not reveal significant differences across the four groups (df = 3, *F* = 1.903, *p* = 0.129).

#### Psychophysiological interaction analysis

Psychophysiological interaction analysis was conducted using SPM12 software running under MATLAB 8.6. Firstly, the left and right MDN were selected as regions of interest and anatomically identified using the Talairach Daemon Brodmann Areas + atlas on WFU_PickAtlas 3.0.5 software^23^. The left MDN centered on (−6.15, −17, 7.85; MNI co-ordinates) and the right MDN centered on (6.31, −17, 7.85). The raw mean time-series of voxels in each region of interest were then extracted for each subject using the MarsBaR toolbox^24^ for SPM. For each subject, the extracted values were correlated with all other voxels in the brain using linear regression with the subject head motion parameters modeled as co-variates of no interest. Subject-level t-contrast maps were combined into a group-level analysis of variance (ANOVA) with age and gender modeled as covariates of no interest. Effectively, this seed-based approach conforms to a psychophysiological interaction analysis^25^. In other words, we use the regression slope of activity in any given voxel on activity in the (thalamic) seed region as a measure of direct (linear) coupling and then compared this measure between diagnostic groups. This psychophysiological interaction analysis has the advantage of not conflating coupling with the amplitude of error terms—a recognized methodological issue in the statistical analysis of (Z-transformed) functional correlation measures^25^.

Alterations specific to schizophrenia were identified by comparing this group against the other three groups (e.g., schizophrenia vs. HC, PTSD, and MDD) and then using the inclusive masking option (at *p* < 0.05 uncorrected) in SPM12 software to identify those regions that survived the individual comparisons (i.e., schizophrenia vs. HC; schizophrenia vs. PTSD; schizophrenia vs. MDD). Here the use of inclusive masking enabled a simple form of conjunction analysis that allowed us to identify alterations specific to the schizophrenia group. Alterations common to the three diagnostic groups were identified by comparing schizophrenia, PTSD, and MDD patients against HC (e.g., schizophrenia, PTSD and MDD vs. HC) and then using the inclusive masking option (at *p* < 0.05 uncorrected) in SPM12 software to identify those regions that survived the diagnosis-specific comparisons (i.e., schizophrenia vs. HC; PTSD vs. HC; and MDD vs. HC). Here the use of inclusive masking enabled us to detect alterations in schizophrenia group that reflect a trans-diagnostic feature of psychiatric illness. Statistical inferences were made at *p* < 0.05 after family-wise error (FWE) correction with a minimum extent threshold of 5 voxels.

## Results

### Thalamic effective connectivity alterations specific to schizophrenia

**Fig. 1:**
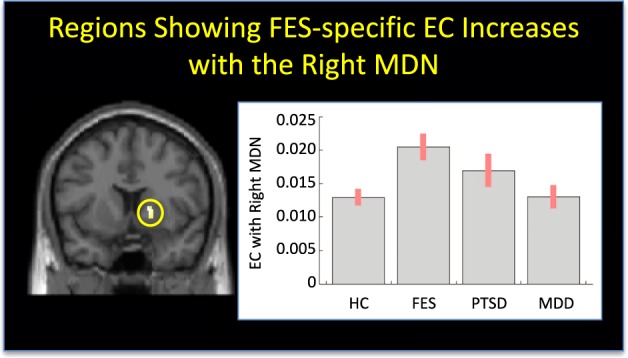
Region showing schizophrenia-specific effective connectivity increases with the right MDN. Left: region of the right pallidum showing EC increases with the right MDN in the schizophrenia group relative to the HC, PTSD and MDD groups. Right: mean right MDN-right pallidum effective connectivity for the FEP, MDD, PTSD, and HC groups (standard error in brackets). FES, first episode schizophrenia, PTSD, post-traumatic stress disorder, MDD, major depressive disorder, HC, healthy controls

For the left MDN, no regions exhibited significant schizophrenia-specific EC alterations. In contrast, the right MDN displayed schizophrenia-specific increased EC with the right pallidum (MNI co-ordinates: *x* = 15 *y* = 5 *z* = 4; *Z*-score = 5.23 *p*-value after FWE correction = 0.018, cluster size: 9 voxels; see Fig. 1).

### Trans-diagnostic thalamic effective connectivity alterations

**Fig. 2:**
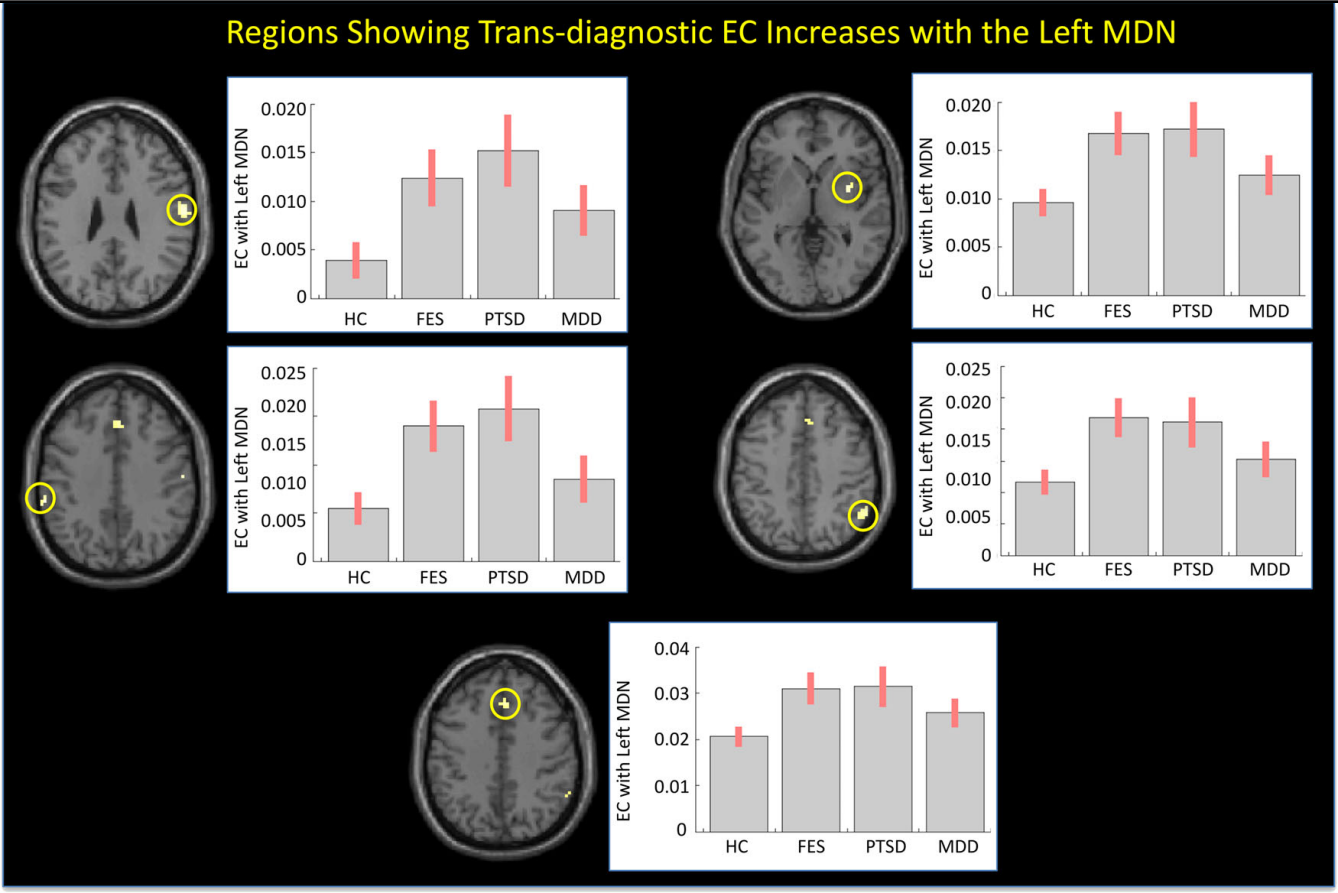
Regions showing trans-diagnostic effective connectivity increases with the left MDN. Left: Regions showing EC increases with the left MDN in the schizophrenia, PTSD, and MDD groups relative to the HC group. Right: mean effective connectivity for each group (standard error in brackets). FES, first episode schizophrenia; PTSD, post-traumatic stress disorder; MDD, major depressive disorder; HC, healthy controls

The left MDN displayed significant trans-diagnostic EC increases with five regions including the right postcentral gyrus, left supramarginal gyrus, right putamen, right supramarginal/angular gyrus and right medial superior frontal gyrus (*p* < 0.05, FWE corrected, see Table 2; Fig. 2).

**Table 2:** Regions showing trans-diagnostic effective connectivity increases with the left medio-dorsal thalamic nucleus

Region	Hemisphere	Co-ordinates (MNI)			Cluster size	*Z* score	*p* value (FWE corrected)
		*X*	*Y*	*Z*			
Postcentral gyrus	Right	60	−10	31	59	5.05	0.002
Supramarginal gyrus	Left	−60	−34	37	9	4.84	0.019
Putamen	Right	33	2	1	9	4.66	0.019
Supramarginal/Angular gyrus	Right	57	−52	43	11	4.63	0.016
Medial superior frontal gyrus	Right	3	29	40	16	4.58	0.012

**Fig. 3:**
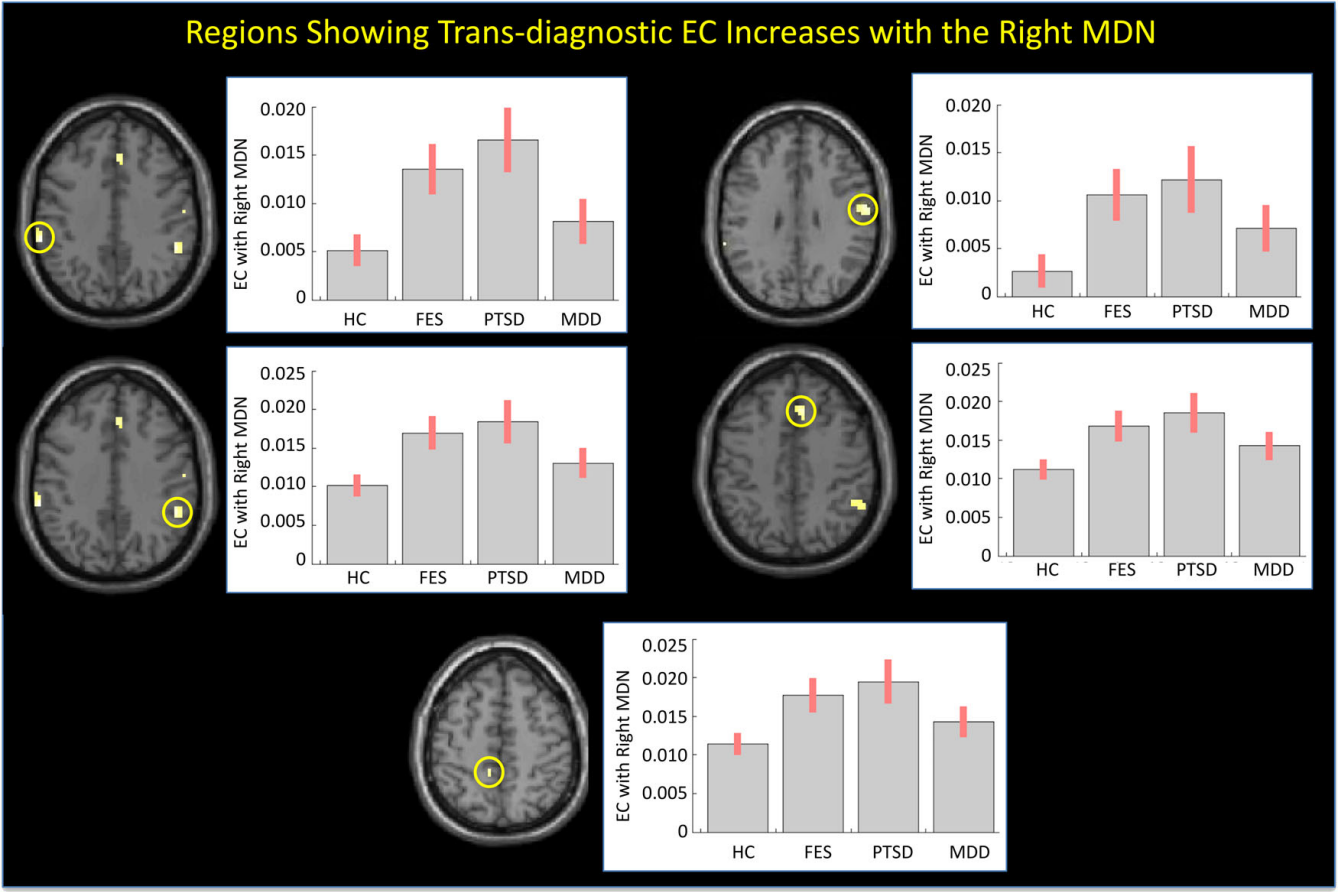
Regions showing trans-diagnostic effective connectivity increases with the right MDN. Left: Regions showing EC increases with the right MDN in the schizophrenia, PTSD, and MDD groups relative to the HC group. Right: mean effectivity connectivity for each group (standard error in brackets). FES, first episode schizophrenia; PTSD, post-traumatic stress disorder; MDD, major depressive disorder; HC, healthy controls

In addition, the right MDN displayed significant trans-diagnostic EC increases with five significant regions including the bilateral supramarginal gyrus, right postcentral gyrus, right medial superior frontal gyrus and left middle cingulate cortex (*p* < 0.05, FWE corrected, see Table 3; Fig. 3).

**Table 3:** Regions showing trans-diagnostic effective connectivity increases with the right medio-dorsal thalamic nucleus

Region	Hemisphere	Co-ordinates (MNI)			Cluster size	*Z* score	*p* value (FWE corrected)
		*X*	*Y*	*Z*			
Supramarginal gyrus	Left	−60	−37	37	14	5.17	0.012
Supramarginal gyrus	Right	51	−43	37	31	4.91	0.005
Postcentral gyrus	Right	60	−10	31	28	4.83	0.005
Medial superior frontal gyrus	Right	9	26	43	28	4.80	0.005
Middle cingulate cortex	Left	−9	−40	52	8	4.67	0.019

## Post hoc correlation analysis in schizophrenia

To further elucidate the above alterations, a series of exploratory post hoc correlation analyses were performed with clinical scores in patients with schizophrenia using IBM SPSS version 21.0 (IBM Corp, Armonk, NY, USA). This identified three significant correlations with clinical scores. First, the trans-diagnostic EC increase between the left MDN and the right medial superior frontal gyrus was negatively correlated with the PANSS negative score (Pearson correlation = −0.328; two-tailed *p*-value: 0.020). Second, the trans-diagnostic EC increase between the left MDN and the right putamen was positively correlated with the Global Assessment of Functioning scores (Pearson correlation = 0.389; two-tailed *p*-value: 0.005). Third, the trans-diagnostic EC increase between the right MDN and the right medial superior frontal gyrus was negatively associated with the PANSS general psychopathology score (Pearson correlation = −0.323; two-tailed *p*-value: 0.022).

## Discussion

The aim of this study was to differentiate between alterations in MDN EC patterns that are a specific feature of schizophrenia and trans-diagnostic alterations that can also be found in other psychiatric illnesses including MDD and PTSD. By investigating medication-naïve participants experiencing their first episode of schizophrenia, we were able to assess thalamocortical dysconnectivity without confounds of antipsychotic medication and prolonged exposure to the illness. In particular, we focused on the MDN—a section of the thalamus that has been found to be functionally impaired in previous task-based^8^ and resting-state^9^ studies of psychosis.

Our first finding of note was increased EC between the right MDN and the right pallidum in the schizophrenia group relative to the HC, PTSD and MDD groups. Although our psychophysiological interaction analysis would suggest greatest sensitivity of the pallidum to thalamic activity in the schizophrenia group, a complementary interpretation is that the sensitivity of the thalamus to afferents from the pallidum was reduced; indeed this is the preferred interpretation given the prevalence of polysynaptic projections from the pallidum to the thalamus (as opposed to the reciprocal direction)^26^. Exploration of the group-specific parameters revealed that, although the EC between the right MDN and the right pallidum was greatest in the schizophrenia group suggesting some diagnostic specificity, a non-significant trend was also evident in the PTSD group relative to the HC and MDD groups (Fig. 1).

Multiple lines of evidence implicate the pallidum in schizophrenia^27^. For example, gray matter volume in this region has been found to be positively related with genetic risk for the illness^28^, whilst abnormal resting-state interhemispheric EC between left and right pallidum has been shown to be associated with negative symptoms, illness duration, and cognitive impairments in patients^29^. In the present study, however, there was no statistically significant association between the strength of the right MDN-right pallidum EC and clinical variables. Based on the increasing evidence for a role of the pallidum in cognition^30^, we speculate that the increased EC between the right MDN and right pallidum could potentially underlie the widely reported cognitive deficits in schizophrenia^31^; this interpretation is consistent with the diagnostic specificity of such deficits that tend to be minimally present in PTSD and MDD^32^.

Our second finding of note was widespread trans-diagnostic EC increases in MDN. Specifically, the left MDN displayed significant trans-diagnostic EC increases with the right postcentral gyrus, left supramarginal gyrus, right putamen, right supramarginal/angular gyrus, and right medial superior frontal gyrus, whereas the right MDN displayed significant trans-diagnostic EC increases with the bilateral supramarginal gyrus, right postcentral gyrus, right medial superior frontal gyrus, and left middle cingulate cortex. The finding of widespread trans-diagnostic increases, observed above and beyond traditional diagnostic boundaries, is potentially consistent with emerging conceptualizations of mental illness based on a single general psychopathology factor^14^.

The observation of increased EC between the bilateral MDN and the right postcentral gyrus during the resting state is consistent with the results of several previous studies of psychosis^3,4,9,33,34^. In particular, it has been suggested that impaired inhibitory interactions between the MDN and primary somatosensory cortex (i.e., the postcentral gyrus) may result in aberrant processing of somatosensory information^35^. Here, we speculate that the increased EC between the bilateral MDN and right postcentral gyrus could reflect altered sensory processing in schizophrenia, PTSD, and MDD, contributing to the emergence of hallucinations/delusions^35^, hyperarousal^36^, and anhedonia^37^, respectively. It is interesting to note that, though previous studies report positive correlations between increased thalamus-somatosensory cortex EC and psychotic symptoms^4,33,38^ no such correlations were found in the present investigation.

The observation of increased EC between the bilateral MDN and the right medial superior frontal gyrus is also consistent with a previous report of increased EC between the thalamus and medial prefrontal cortex (which includes the medial superior frontal gyrus) in schizophrenia^39^. This finding could reflect a compensatory response to possible regional structural^40^ and functional^41^ deficits in the medial superior frontal gyrus that are typically observed in this illness. Interestingly, the EC increase between the left MDN and the right medial superior frontal gyrus was negatively correlated with the PANSS negative score, while the EC increase between the right MDN and the right medial superior frontal gyrus was negatively associated with the PANSS general psychopathology score. These negative correlations support our speculation of the present findings representing compensatory responses to the illness.

In addition, we found that the right MDN displayed increased EC with the bilateral supramarginal gyri, whilst the left MDN exhibited this same pattern with the left supramarginal gyrus. With the supramarginal gyrus being thought to function in the phonological loop component of working memory^42^, the increased EC between the MDN and the supramarginal gyri could be associated with impaired verbal working memory processing in schizophrenia, PTSD, and MDD subjects, possibly with regards to a compensatory response.

We also found a number of effects which were lateralized to the left or right MDN. In particular, the right MDN displayed increased EC with the left middle cingulate cortex. The latter is thought to play a key role in attention and awareness—areas of cognitive functioning that are impaired across psychiatric disorders. Therefore, increased EC between this region and the MDN could be associated with impairments of attention and awareness. In addition, the left MDN showed increased EC with the right putamen—a finding which is inconsistent with previous studies reporting decreased EC between the MDN and the putamen in chronic schizophrenia^7,9^ and in an at risk mental state for psychosis^43^. Interestingly, the EC increase between the left MDN and the right putamen was positively correlated with the Global Assessment of Functioning scores. The finding that schizophrenia patients with relatively higher EC values between these regions had a greater day-to-day functioning suggests that this effect might also represent a compensatory response to the illness.

A strength of the present investigation is that, at the time of scanning, patients with schizophrenia were experiencing their first episode of the illness and were still medication-naïve. This means that the effects reported here cannot be explained by illness chronicity or medication—two common confounds in neuroimaging studies of psychiatric disease. This absence of chronicity, and medication-related effects may account for the fact that, in the present investigation, we found increases in thalamocortical EC whereas previous studies employing the same resting-state paradigm tended to report decreases. We speculate that some of the decreases reported in the existing literature may reflect the effects of these two common confounds. A further strength is that all four groups were scanned using the same MRI scanner and image acquisition parameters over the same period of time, and therefore our results cannot be explained by systematic differences in the acquisition of the data.

In addition, the present investigation has several limitations that are worth mentioning. Firstly, we cannot exclude the possibility that the increased EC between the right MDN and the right pallidum that we report as a distinctive feature of schizophrenia may be expressed in other psychiatric groups not included here. For example, a recent investigation reported divergent patterns of thalamocortical dysconnectivity in patients with schizophrenia and bipolar disorder^44^. However, a direct comparison with our findings is difficult due to major methodological differences between the two studies. For example, we focussed on a specific nucleus of the thalamus (the MDN) whereas Skåtun and colleagues examined ten sub-sections of the thalamus that were identified using independent component analysis rather than pre-existing neuroanatomical knowledge. Furthermore we examined drug-naïve patients with a first episode of the illness, whereas Skåtun and colleagues recruited medicated patients who had been ill for an average of 5.8 years (for schizophrenia) and 9.9 years (for bipolar disorder). Secondly, although we can exclude medication as a potential confound, there are other sources of neurofunctional variability such as IQ and socio-economic status that were not monitored and might have contributed to the differences between patients and controls. We also note that there were demographic differences amongst clinical groups—for example the PTSD participants came from rural rather than urban areas and were older than the other groups. These differences however cannot account for our main finding, i.e., the presence of trans-diagnostic EC alterations between the MDN and the cortex in all clinical groups. Thirdly, because the data were acquired using a cross-sectional rather than a longitudinal design, it was not possible to distinguish between correlates of illness vulnerability and correlates of illness onset. Fourthly, our results are based on the MDN and may not be relevant to other sections of the thalamus, which is both structurally and functionally heterogeneous^3^.

In conclusion, the present study found limited evidence for schizophrenia-specific alterations, indicating that dysconnectivity of the MDN is not a unique feature of schizophrenia. Instead, we found a number of trans-diagnostic alterations, indicating that EC alterations between the MDN and the cortex are primarily a trans-diagnostic feature of psychiatric disorders—compatible with emerging conceptualizations of mental illness based on a single general psychopathology factor^14^. Therefore, the EC of MDN could potentially be used to assess the presence and progression of general psychopathology above and beyond traditional diagnostic boundaries.
